# Evaluation of Potentially Toxic Elements in Roadside Agricultural Soils Using Pollution Indices and Remediation Potential of Manure and Attapulgite in Wheat Cultivation

**DOI:** 10.3390/toxics14060483

**Published:** 2026-05-31

**Authors:** Apostolia Argiri, Aikaterini Molla, Miltiadis Tziouvalekas, Christina Emmanouil

**Affiliations:** 1 Secondary Education of Thessaly, Ministry of Education, Religious Affairs and Sports, 38333 Volos, Greece; apoargyri@sch.gr; 2 Ministry of Rural Development and Food, 11143 Athens, Greece; 3 Institute of Industrial and Forage Crops, Hellenic Agricultural Organization—DEMETER, 41335 Larissa, Greece; mtzouvalekas@gmail.com; 4 School of Spatial Planning and Development, Aristotle University of Thessaloniki, 54124 Thessaloniki, Greece

**Keywords:** potentially toxic elements (PTE) contamination, risk assessment, wheat cultivation, attapulgite, biological concentration factor, coefficient of contamination level

## Abstract

Soil near urban areas may be burdened with numerous environmental pollutants including potentially toxic elements (PTEs). In this context, samples near the highway infrastructure in Larissa, Central Greece were examined for pseudo-total concentrations of Cr, Cu, Zn, Pb and Ni, and enrichment, ecological risk and human risk indices were calculated. Co-variation structure between PTEs and key soil properties was assessed through Principal Component Analysis (PCA). Screening for the pollution status of this area would quantify the possible risk, and therefore whether our subsequent rehabilitation trials would be of use. In this context, the most polluted sample was chosen to undergo a variety of remediation alternatives in a pot experiment, incorporating wheat and manure–attapulgite mixtures. Results showed enrichment of soil mainly with Ni, a low probability (9%) of risk exceedance for children for non-carcinogenic health effects and strong associations between the PTEs, indicating common sources. The greenhouse experiments showed that the application of manure–attapulgite reduced PTE concentrations in soil and wheat plant, with the greatest decrease observed for Pb, Cr and Ni. BCF values indicated strong accumulation of Ni (BCF > 1), while Cr and Cu showed limited uptake. Coefficient of contamination level (CCL) values (<1) for Cr and Cu confirmed reduced plant uptake, whereas Ni, Pb and Zn remained above 1. Taken together, the research shows that the fields chosen here are subjected to significant PTE input from lithogenic and anthropogenic sources, which may even become dangerous for sensitive sub-populations. Experimental cultivation of wheat shows that the combined amendments effectively reduced metal bioavailability and soil-to-plant transfer.

## 1. Introduction

Roadside soil is increasingly recognized as important sinks for PTEs, primarily due to continuous inputs from vehicular traffic and other urban and industrial activities [1,2,3]. Atmospheric deposition and rain-induced wash-off may also contribute through particle transport, deposition and hydrologically driven redistribution [4,5,6]. Emissions from fuel combustion, tire and brake wear, lubricating oils and road surface degradation are significant factors for enrichment with chromium (Cr), copper (Cu), nickel (Ni), lead (Pb) and zinc (Zn) in soils to road networks [7,8,9]. Over time, these contaminants may persist in the soil environment, posing risks to ecosystems, agricultural productivity and human health through plant uptake and entry into the food chain [10,11].

The assessment of soil contamination using pollution indices has become a widely adopted approach for evaluating the extent, sources and ecological risks of PTEs [12,13]. Indices such as contamination factor (CF), Geo-accumulation Index (Igeo), Pollution Load Index (PLI), Nemerow Pollution Index (NI) and Improved (modified) Nemerow index (INI) provide valuable insights into the degree of contamination and help distinguish between geogenic and anthropogenic contributions [14,15,16]. In Thessaly, in particular, Skordas and Kelepertsis [17] reported elevated Ni and Cr concentrations in agricultural soils and attributed them mainly to lithogenic influences associated with ultrabasic rocks.

Human health risk indices are tools used for assessing the adverse effects from exposure of human populations to PTEs. These indices integrate contaminant concentrations with exposure pathways (ingestion, inhalation and dermal contact) to estimate non-carcinogenic and carcinogenic risks [18,19]. Commonly applied metrics include the Hazard Quotient (HQ), Hazard Index (HI) and Cancer Risk (CR), which enable the quantitative evaluation of health risks associated with contaminated soils [20,21]. Such approaches are particularly important for identifying vulnerable populations, especially children, who are more susceptible to toxic exposures [14,20]. By providing a standardized framework, human health risk indices support environmental management, remediation planning and policy-making aimed at minimizing the impacts of soil contamination on human health [22,23].

Organic amendments, such as manure, have been shown to improve the soil physicochemical properties, including soil structure, water-holding capacity, cation exchange capacity and improve overall soil fertility. In addition, manure plays a crucial role in the immobilization of PTEs through several mechanisms, including adsorption onto organic matter surfaces and precipitation reactions that reduce metal solubility and bioavailability [24,25]. These processes lead to decreased mobility and plant uptake of metals, contributing to the mitigation of environmental and human health risks [25,26]. Moreover, manure is considered a cost-effective and sustainable strategy for contaminated soils, particularly when applied alone or in contamination with mineral amendments [25,26].

Mineral amendments like attapulgite have gained increasing attention as effective materials for the remediation on contaminated soils due to their unique properties [27,28]. Attapulgite is a naturally clay mineral characterized by specific surface area, porosity and cation exchange capacity, which enable strong adsorption of PTEs [20,29]. Its structure contains abundant silanol and aluminol functional groups that facilitate metal binding through ion exchange, surface complexation and electrostatic interactions [30]. Additionally, attapulgite can promote the precipitation of metals as stable mineral phases, thereby reducing their mobility and bioavailability in soils [20,31] [20,30]. Moreover, attapulgite is environmentally friendly, widely available and cost-effective, making it a promising material for sustainable soil remediation [31].

The co-application of organic and mineral amendments has been recognized as an efficient approach for the remediation of contaminated soils. Their synergistic interaction can further reduce metal mobility through multiple mechanisms, including adsorption, complexation and precipitation processes. As a result, the co-application of manure and attapulgite not only improves soil physicochemical properties but also enhance remediation efficiency, making it an effective approach to soil contamination [32].

Wheat (*Triticum durum*), as one of the most widely cultivated cereal crops globally, is particularly relevant in this context due to its importance in human nutrition and its potential to accumulate PTEs from soils contaminated with PTEs. Therefore, understanding the interactions between soil amendments and PTE dynamics in wheat cultivation systems is critical for ensuring food safety and sustainable agricultural practices [33,34].

Studies have investigated the use of soil amendments in wheat cultivation system to reduce PTE uptake [20,35]. Similarly, mineral amendments such as attapulgite have been reported to reduce bioavailability by stabilizing contaminants in less mobile forms, thereby lowering plant uptake [20]. A recent study demonstrated that soil amendments improve soil physicochemical properties and microbial activity, reducing the accumulation of toxic elements in wheat grains and contributing to safer agricultural production [25].

In this context, the present study aims to (i) screen for PTE contamination in agricultural fields near roadside using pollution and risk indices, focusing on a crop-producing area in mainland Greece and (ii) investigate the effectiveness of manure and attapulgite, in reducing PTEs availability and uptake in wheat cultivation, one of the staple cereals in Europe [36]. This study addresses a widespread and growing environmental issue: the accumulation of PTEs in agricultural fields near intensive human activity. The work also advances practical remediation strategies by demonstrating the effectiveness of sustainable, low-cost amendments such as manure and attapulgite, applied individually and in combination. The findings highlight how synergistic approaches can reduce metal mobility and bioavailability, ultimately limiting their transfer into crops.

## 2. Materials and Methods

### 2.1. Study Area

**Figure 1:**
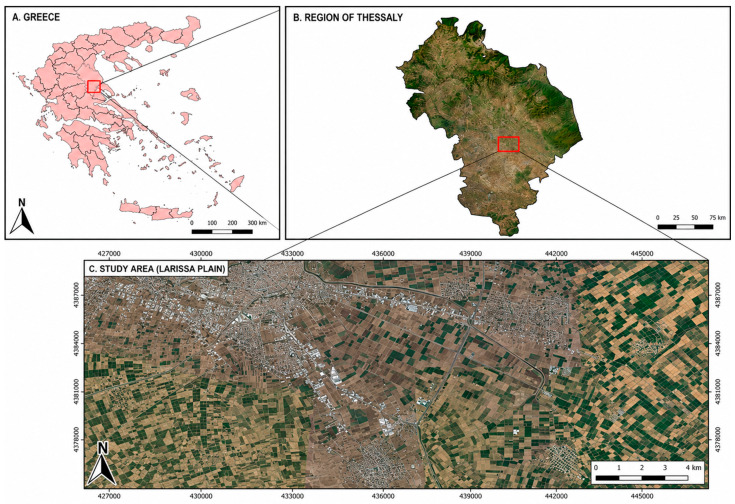
Location of the study area (Larissa, Greece).

The study area is situated near the city of Larissa, where the new national highway and the old national road connect Larissa to Athens and Larissa to Volos, respectively. Most of the sampled soils were collected from cultivated fields located in close proximity to these highways (Figure 1). Additionally, within the study area there is an industrial facility involved in the production of aluminum and other metal products and may represent a localized anthropogenic source of PTEs.

### 2.2. Soil Sampling

**Figure 2:**
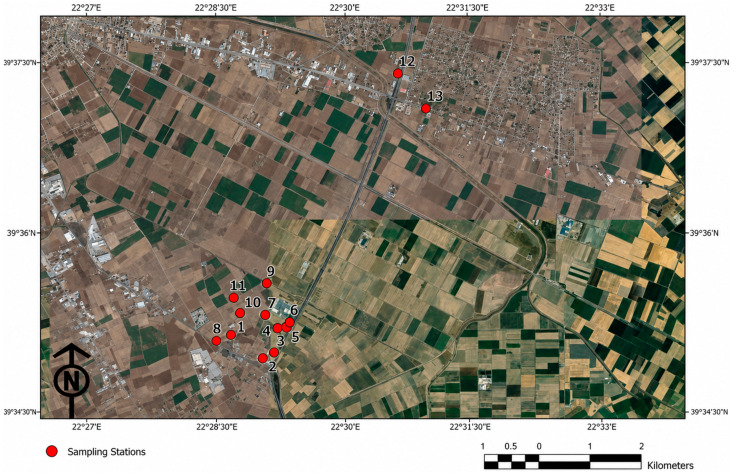
Distribution of the soil sampling points in the study area.

A total of 13 soil samples were collected from the studied area. Three subsamples were obtained from each soil sample and then the subsamples were mixed into a single composite sample. All the soil samples were obtained using a steel sampling tool from a depth of 0–30 cm. The geographic coordinates (latitude and longitude) of each soil sampling point were recorded using a portable GPS and digital maps were created using the QGIS 4.0 program (Figure 2).

Sampling points were distributed across cultivated agricultural fields adjacent to the highway network in order to achieve representative spatial coverage of the study area and to capture possible variations in PTE accumulation associated with traffic and nearby industrial activities. Site selection was additional based on field accessibility and land use characteristics. Composite sampling was applied at each point to minimize local heterogeneity.

### 2.3. Soil Samples’ Analysis

The collected soil samples were first air-dried at 60 °C in a forced-draft oven (L101-0AB, Chemist EU, Niel, Belgium) until constant weight was achieved and then crushed using a ceramic mortar. Afterwards each soil sample was passed through a 2 mm sieve. The physicochemical properties which were determined in each sample were pH in a 1:1 (soil:distilled water) ratio (C520 Multi-Parameter Analyzer, Consort, Turnhout, Belgium), Electrical conductivity (EC) in a 1:5 (soil:distilled water) ratio (Seven Excellence S470, Mettler Toledo, Greifensee, Switzerland), calcium carbonate (CaCO_3_) content using the Bernard calcimeter (Gabbrielli Technology, Calenzano, Italy) and organic matter (OM) content was assessed by the Walkley–Black wet oxidation method with 0.17 N potassium dichromate (K_2_Cr_2_O_7_), following by back-titration with 0.5 N FeSO_4_. Moreover, particle size distribution (sand, silt, clay) was evaluated using the Bouyoukos method. All analyses were conducted according to the methodology described by Rowell (2014) [37]. Additionally, the pseudo-total concentration of the studied PTEs (Cr, Cu, Zn, Pb, Ni) was determined. The digestion procedure protocol was carried out over a period of 5 days. On the 1st day, each sample (approximately 1 g, accurately weighted) was transferred into a 50 mL Erlenmeyer flask. Subsequently, HNO_3_ (65% *w*/*w*, 5 mL) was added and the samples were left undisturbed at room temperature for 24 h without heating. On the 2nd day, the samples were heated in a sand bath at 85–95 °C for 4–5 h with temperature monitored throughout the process. On the 3rd day, HNO_3_ (5 mL) was added, followed by further heating at 85–98 °C for another 4–5 h. On the 4th and 5th days, H_2_O_2_ (30% *w*/*w*, 3 mL) was added. At the end of the digestion process (5th day), the samples were filtered and transferred into 50 mL volumetric flasks. All analyses were performed in triplicate of each sample. The reagents used were of analytical grade [14]. The pseudo-total concentrations of the PTEs in the digestates were determined employing flame AAS (FAAS) model Thermo 3000 series (Thermo Fisher Scientific, Waltham, MA, USA). All analyses were performed in triplicate. Calibration was performed using multi-element standard solutions, and instrument performance was routinely verified with quality control standards. Limits of detection (LOD) and limits of quantification (LOQ) are shown in Table S1. Method accuracy was assessed through analysis of certified reference materials (WEPAL-QUASIMEME, Wageningen University, Wageningen, The Netherlands), with recoveries ranging between 93.5–98.1%.

### 2.4. Greenhouse Experiment

#### 2.4.1. Experimental Design

According to the results of the soil samples studied, the soil sample with the highest PTEs concentration was used to conduct the pot experiment in the greenhouse. The selected soil belonged to a field located near the new highway in Larissa region. A quantity of ca. 50 kg soil from the selected field was collected from a depth of 0–30 cm. The attapulgite that was used for the experiments (AGLEV 200, Plisiotis Nikolaos, Larissa, Greece) is characterizes by a high cation exchange capacity (100–200 cmol kg^−1^), slightly alkaline pH (7–8.5) and a porous aluminosilicate structure rich in SiO_2_ (60–70%) and Al_2_O_3_ (10–15%). It exhibits high water retention capacity and ion-exchange properties due to its microporous framework, with pore sizes typically in the range of 0.3–0.8mm.

Treatments included a mixture of manure (Plisiotis Nikolaos, Larissa, Greece) and attapulgite and applied in soil at three different levels (0%, 3% and 4% mixture of manure—attapulgite/kg soil). For the experiments in greenhouse, wheat (*Triticum durum*) was used. Each treatment comprised three replicates and the pots were arranged in a randomized design. The treatments are presented in Table 1. A total of 21 pots were placed in the greenhouse and the pot experiment lasted for 4 months, from 1 November 2020 to 30 April 2021. An automatic drip irrigation system was used for irrigation. The pots were watered to maintain soil moisture at 65% of water-holding capacity using the gravimetric (weighing) method to ensure adequate water supply for wheat growth. Plants were harvested on day 120 post seed sowing.

#### 2.4.2. Soil, Manure and Plant Analysis of the Collected Field

The physicochemical characteristics of the soil and manure were determined following the same methodologies as described above (Section 2.3) Furthermore, the PTEs concentrations were quantified as described above. The plant tissue of each plot was harvested, washed with HCl 1 M followed by deionized water and dried at 70 °C for 48 h. Then, the biomass was weighed, ground into a fine powder and stored in plastic bags. For the total determination of Pb, Ni, Cu, Cr and Zn content, 1 g of plant material was ashed at 520 °C for 4 h in porcelain crucibles. The ash of each plant tissue was treated with 20 mL of 20% HCl and the extracts were analyzed using flame AAS (FAAS) model Thermo 3000 series (Thermo Fisher Scientific, Waltham, MA, USA) [38,39,40].

### 2.5. Assessment of Indices of Contamination

#### 2.5.1. Contamination Factor (CF)

To evaluate the extent of soil contamination and to distinguish between geogenic and anthropogenic in puts of PTEs, the CF was calculated as follows proposed by Hakanson [41]:

(1) $$CF=\frac{C_{sample}}{C_{background}}$$

where C_sample_ denotes the content of PTEs in the soil sample, C_background_ refers to the corresponding background concentration. In the absence of site-specific background values, the universal values from Kabata-Pendias [42] were used and are listed in Table 2. The results were critically assessed according to Skordas and Kelepertsis [17], who also compared agricultural fields in Thessaly plain to global backgrounds. The contamination levels of CF are illustrated in Table S2.

#### 2.5.2. Pollution Load Index (PLI)

The PLI is used to evaluate the overall level of contamination by considering the combined effect of multiple PTEs. It is calculated as the mean of the CF of all analyzed PTEs [43].

(2) $$PLI={\left(CF_{Cr}\times CF_{Ni}\times CF_{Cu}\times CF_{Zn}\times CF_{Pb}\right)}^{1}/n$$

where CF refers to the contamination factor of the studied PTEs and n denotes the total number of analyzed PTEs (*n* = 5). The classification categories of PLI are presented in Table S3.

#### 2.5.3. Geo-Accumulation Index (Igeo)

The Igeo is widely used to assess PTE contamination in soils, as it reflects both the influence of anthropogenic activities and the environmental impact of PTEs [44]. The index was introduced by Müller [45] and the following equation was used for its determination:

(3) $$I_{geo}=\log_{2}(\frac{C_{n}}{1.5\times B_{n}})$$

where Cn represents the concentration of the nth PTE and Bn denotes its corresponding background value. The factor 1.5 is included to account for possible variations in background levels due to lithogenic effects. The classification categories of Igeo are provided in Table S4.

#### 2.5.4. Nemerow Pollution Index (NI) and Improved (Modified) Nemerow Index (INI)

The NI is widely used for the assessment of soil quality, as it emphasizes the contribution of the PTE exhibiting the highest CF. This index was calculated using the following equation [46]:

(4) $$NI=\frac{\sqrt{{CFmean}^{2}+{CFmax}^{2}}}{\surd 2}$$

where CFmean represents the average value of CF for all analyzed PTEs and CFmax corresponds to the maximum CF among them.

The INI provides a more accurate representation of soil contamination by incorporating alternative pollution indices, such as the Igeo instead of CF, and is measured as follows [47]:

(5) $$INI=\frac{\sqrt{{Igeomax}^{2}+{Igeoave}^{2}}}{\surd 2}$$

where: Igeomax is the maximum value of the Igeo values and Igeoave is the average value of the Igeo values calculated for all the investigated PTEs.

The classification categories of NI and INI are summarized in Tables S5 and S6, respectively.

### 2.6. Assessment of Biological Concentration Factor (BCF) and Coefficient of Contamination Level (CCL)

The accumulation of PTEs from soil to plant was assessing using the BCF [48]. The BCF is used to evaluate the ability of plants to accumulate the PTEs from soil into edible parts of plants. This indicator is useful for assessing the safety of crops cultivated in contaminated environments. The BCF is calculated as follows:

(6) $$BCF=\frac{CPTEp}{CPTEs}$$

where CPTEp is the PTE concentration in the plant of wheat, and CPTEs is the PTE concentration in the soil (mg/Kg dry weight). A BCF value greater than 1 indicates metal accumulation in plant tissue, values close to 1 suggest limited influence of the metal on the plant, while values below 1 reflect no metal uptake to plant issue [49].

Furthermore, the coefficient of contamination level (CCL) was calculated according to the following formula [50]:

(7) $$CCL=\frac{PTE\;in\;plant\;in\;amended\;treatment}{PTE\;in\;plant\;in\;control\;treatment}$$

The CCL is the ratio of PTE concentration in plant tissue cultivated at PTE-contaminated pots to PTE concentration in plant tissue cultivated at control pots. Values of CCL lower than 1 indicate reduced metal uptake due to amendment application, reflecting decreased metal bioavailability, whereas values greater than 1 suggest enhanced uptake [50].

### 2.7. Assessment of Human Health Risk Indices

#### 2.7.1. Hazard Quotient (HQ) and Hazard Index (HI)

The possible effects to human health from contact with the polluted soils are calculated here. The present methodology is outlined in [51]. The subsequent refinements and changes found either in USEPA-issued guidance documents or in other reliable sources up to 2026 have been included.

The average daily intake dose (ADD, mg/kg/day) of PTEs through ingestion (ADDing), dermal contact (ADDderm) and inhalation (ADDinh) for children and adults were determined using the following formulas:

(8) $$ADD_{iing}=\frac{C_{i}\times \;IR_{ing}\times \;EF\;\times \;CF\;\times \;ED}{BW\;\times \;AT}$$

(9) $$ADD_{iinh}=\frac{C_{i}\times IR_{inh}\times EF\times ED}{PER\times BW\times AT}$$

(10) $$ADD_{iderm}=\frac{C_{i}\times SA\times CF\times AF\times ABS\times EF\times ED}{BW\times AT}$$

where C_i_ = the concentration of the PTE_i_ in the soil; IR_ing_ = the ingestion rate; IR_inh_ = the inhalation rate; EF = the exposure frequency; BW = the average body weight; CF = the conversion factor; ED = the exposure duration; SA = the exposure skin area; AF = the soil to skin adsorption coefficient; PEF = the surface dust emission factor; ABS = the dermal absorption factor; and AT = the average action time.

The non-carcinogenic risk index (HQ) and the total non-carcinogenic risk index (HI) were calculated according to the following:

(11) $$HQ=\;\frac{ADDi}{RfDi}$$

(12) $$HI=\sum HQ_{i}=\sum \left(H{IQ}_{ing}+H{IQ}_{inh}+H{IQ}_{der}\right)$$

#### 2.7.2. Carcinogenic Risk (CR) and Total Carcinogenic Risk (TCR)

Similar methodology was followed for calculating carcinogenic risk. The carcinogenic risk (CR) and the total carcinogenic risk (TCR) were calculated according to the following:

(13) $$CR_{i}=\sum \left(ADD_{i}\;\times \;SF\right)$$

(14) $$TCR=\sum CR_{i}$$

#### 2.7.3. Refinement of Input Parameters and Probabilistic Risk Assessment

##### Body Weight Refinement

Body weight in the initial USEPA risk assessment calculations was equal to 70 kg for an adult and 15 kg for a child. However, these weights may not be representative of current European populations. For refinement for the present set of data for Greek people the following steps were performed:

A Adult body weight was derived from BMI (Body Mass Index) recorded for Greece in 2006, which was 26.5 kg/m^2^ on average [52], and from average height values for Spanish and Italian Europeans (mean value out of two; for men was 1.71 m and for women 1.61 m) [53]. As such, average body weight was 77.5 kg for men and 68.7 kg for women. A conservative estimation of the SD for weight was derived from a large study in 12 countries including Greece [54] and additionally from a Greek study [55], and it was adjusted to 13 kg for both sexes.
B Children body weight (boys and girls) was derived from the growth curves (50th, 95th percentiles) for 6 years old Greek children from [56] and it was equal to 22 and 28 kg respectively.

##### Validation and Attribution of the Remaining Parameters

Remaining parameters and their distributions are shown in Table S7. RfD and SF values used are shown in Table S8. As described there, we adopted a tiered approach with the USEPA IRIS database as the most reliable source. When no value was found in IRIS, the sources mentioned in Table S8 were preferentially chosen.

##### Probabilistic Risk Assessment

A probabilistic risk assessment as recommended by [57] was performed on Crystal Ball software 11.1.2.4 (Oracle Corp., Austin, TX, USA). Instead of one predetermined or mean value, a distribution of values was proposed [58] for various input parameters. The refined parameters and the corresponding statistical probability distributions are shown in Table S7.

### 2.8. Ecological Risk Index Evaluation (Er and PERI)

The potential ecological risk factor, proposed by Hakanson [41], is widely used to assess the extent of PTEs. The PERI includes the single-factor ecological risk index (Er) and the overall ecological risk index (PERI) [59]. The calculation of these indices was performed based on the following formulas:

(15) $$Eri=Tr_{i}\;\times \;CF_{i\;}$$

PERI=∑Eri(16)

where Tr is the toxic response factor for each PTE and CF is the contamination factor (CF). The Tri for the studied PTEs is: Cr = 2, Cu = 5, Ni = 5, Pb = 5 and Zn = 1.

The contamination classes of Er and PERI are summarized in Table S9.

### 2.9. Statistical Analysis of Data

The 13 samples (polluted soils near the highways) were grouped for their parameters PTE log-concentration, pH, EC (μS/cm), CaCO_3_% (*w*/*w*) and OC % (*w*/*w*) utilizing PCA. The suitability of the dataset was assessed using the Kaiser–Meyer–Olkin (KMO) measure and Bartlett’s test of sphericity. A two-factor solution with Varimax rotation was chosen and only loadings above 0.6 were deemed significant. Analyses were performed on SPSS 28 (IBM, Armonk, NY, USA). PTEs in soil and in plant tissue under the different treatments, BCF and CCL were compared between experimental groups using the Statgraphics Centurion software (version 18.1.01; Statgraphics Technologies, Inc., The Plains, VA, USA), with the least significant different (LSD) test performed at a 95% confidence level (*p* < 0.05).

## 3. Results

### 3.1. Results for Physicochemical Properties of the Collected Soil Samples

The range and mean values physicochemical properties of the soil samples from the study area are presented in Table 3. Soil properties showed that pH ranged from 7.1 to 8.1 (mean 7.8) and electrical conductivity (EC) varied between 277 and 662 μS cm^−1^ (mean 488.5 μS cm^−1^). Calcium carbonate (CaCO_3_) content ranged from 1.0 to 5.5% (mean 2.1%) and organic matter (OM) from 1.2 to 2.6% (mean 1.7%). The particle size distribution indicated sand content between 17 and 37%, clay between 17 and 53% and silt between 12 and 45%. Two out of the 13 soil samples were characterized as loamy and the rest of them as clay.

### 3.2. Results for Physicochemical Properties and PTEs of the Soil and the Manure Used for the Greenhouse Experiment

The soil used for the greenhouse experiments was classified as Loam (L), consisting of 38% sand, 45% silt and 17% clay. It was alkaline (pH = 8.21), with a CaCO_3_ and organic matter content of 5.5% and 1.25%, respectively. The concentration of PTEs in the soil were as follows: Pb (9.9 mg kg^−1^), Zn (62.3 mg kg^−1^), Cu (32.5 mg kg^−1^), Ni (159.1 mg kg^−1^) and Cr (180.4 mg kg^−1^).

The physicochemical properties and PTEs of the manure used for the greenhouse experiment were as follows: pH 7.5, 35–45% organic matter (OM) content and 32–42% moisture. The concentration of PTEs were 50 mg kg^−1^ for Cu, 20 mg kg^−1^ for Cr, 15 mg kg^−1^ for Ni, 210 mg kg^−1^ for Zn and 10 mg kg^−1^ for Pb.

### 3.3. Results for Average, Minimum and Maximum PTEs Concentration Results of the Studied Soil Samples

The average, minimum and maximum PTEs content of the 13 studied soil samples are presented in Table 4. According to Table 4, the highest values among the measured PTEs was recorded for Cr (mean value of 126.1 mg kg^−1^) and Ni (mean value of 156.6 mg kg^−1^). Furthermore, Zn content presented a mean value of 60.0 mg kg^−1^, Cu a mean value of 30.1 mg kg^−1^ and Pb a mean value of 10.5 mg kg^−1^.

The concentration of the PTEs could not be compared to local geochemical background because no documented pristine reference soils are known within the study area. Nevertheless, the results were also compared to soils in the cultivated region of Agia in Thessaly area. The concentrations were assessed in comparison with widely recognized soil quality guidelines, including those established by the Canadian Council of Ministers of the Environment [60], the Dutch Target Values [61], the maximum values for biosolid-amended soil according to Greek law [62], the background values by Kabata-Pendias [42] and Haynes [63] and values reported for agricultural soils in Thessaly region by [17]. In the case of Cr and Ni, all the soil samples exceeded the limits of all recognized quality guidelines. In contrast, Cu and Pb values remained below the Canadian limits, the Dutch Target Values, the values set in the Greek law, as well as the values assumed by Kabata-Pendias and by Haynes. In the case of Zn, values were below recognized quality guidelines, while only one soil sample was above when compared with Kabata-Pendias and Haynes limits (concentration > 70 mg kg^−1^). In comparison with agricultural soils from the Thessaly plain reported by Skordas and Kelepertsis [17], the studied soils showed lower levels for all PTEs.

### 3.4. Factor Analysis of Roadside Soil Samples

Regarding the roadside samples PCA analysis, results showed data adequacy (KMO measure= 0.765, Bartlett’s test of sphericity *p* < 0.001). The two-factor solution explained 78.93% of the total variance. Component 1 accounted for 52.95% of the variance and was strongly associated with Zn (0.981), Cu (0.974), Ni (0.942), Pb (0.886), EC (0.765) and Cr (0.739). Component 2 explained 25.98% of the variance and was characterized by positive loadings of pH (0.864) and CaCO_3_ (0.752), and a negative loading of OC (−0.737), reflecting a soil physicochemical gradient.

Given the elevated Ni and Cr concentrations observed in the studied soils, together with previous findings from Thessaly agricultural soils reported by Skordas and Kelepertsis [17], Component 1 likely reflects a mixed lithogenic–anthropogenic signature. The strong co-variation of Ni and Cr may indicate an important contribution from geogenic sources and parent material composition, while traffic-related and industrial inputs may additionally contribute to Zn, Cu and Pb accumulation. On the contrary, Component 2 was mostly highlighting soil properties gradient.

**Figure 3:**
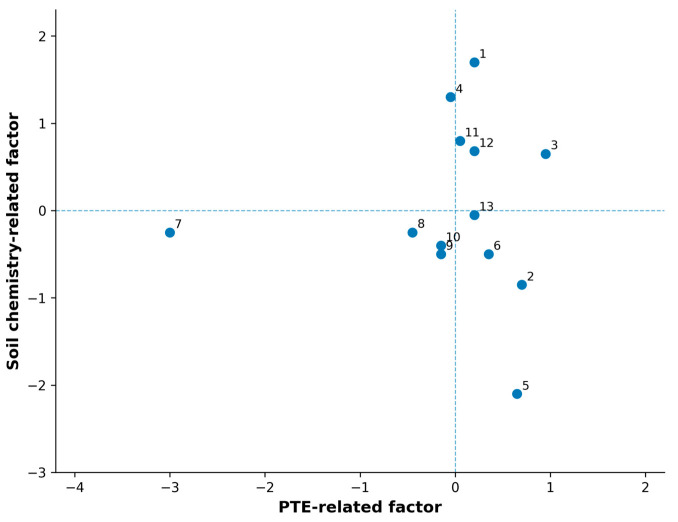
Distribution of roadside samples across PCA biplot.

The distribution of the samples and the loadings of the parameters are shown in Figure 3.

### 3.5. Results for Contamination Factor (CF), Pollution Load Index (PLI) and Geo-Accumulation Index (Igeo) of the Studied Soil Samples

The detailed concentration data of CF and PLI for the investigated PTEs are reported in Table S10. The average, minimum and maximum CF of the 13 studied soil samples are illustrated in Table 5. Ni exhibited the highest CF values, ranging from 4.59 to 5.96, with a mean value of 5.40, classifying it as a considerable level of contamination. Cr showed moderate to considerable contamination, with CF values between 1.10 and 3.03 (mean value 2.12). In contrast, Cu, Pb and Zn presented lower contamination levels, with mean CFs of 0.77, 0.39 and 0.86, respectively. Moreover, as shown in Table S10, 100% of the soil samples are of “considerable contamination” for Ni and 92% of the samples are of “moderate contamination” for Cr. In case of Cu and Zn, 100% of samples showed low contamination levels. Additionally, 92% of samples exhibited low Pb contamination.

The PLI values ranged from 0.755 to 1.387, with a mean value of 1.237. According to the classification levels the area studied was characterized as moderately polluted. Furthermore, according to Table S10, 12 of the 13 samples were classified as moderately polluted.

The detailed values of Igeo for the analyzed PTEs are presented in Table S11, while the mean, minimum and maximum Igeo values are given in Table 6. The results indicated that Cr, Cu and Pb exhibited the lower Igeo values, with mean values −1.15, −0.98 and −1.96, respectively, belonging in Class 0 (uncontaminated). The highest Igeo value was noted for Ni, ranging from 1.6145 to 1.9900 (mean 1.8458), categorizing the samples as moderately contaminated. For Zn, samples were classified as uncontaminated.

Notably, for Ni 100% of the studied soil samples belonged in Class 2 (moderately contaminated) and for Zn 23% of the samples belonged to Class 2 and 77% in Class 1 (from uncontaminated to moderately contaminated).

### 3.6. Results of Nemerow Pollution Index (NI) and Improved (Modified) Nemerow Index (INI) of the Studied Soil Samples

**Figure 4:**
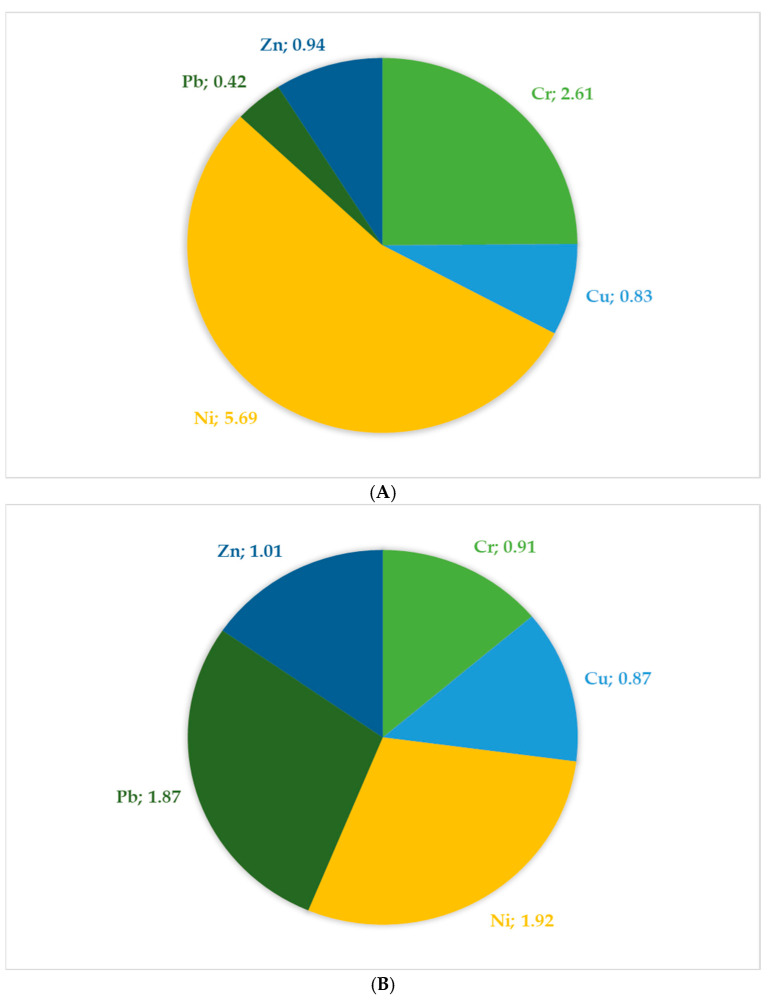
(**A**) Results of the NI and (**B**) results of the INI for each PTE studied.

The NI and INI of the studied soil samples are provided in Figure 4A and Figure 4B, respectively. The results demonstrated that most of the soil samples fall within the moderate pollution category. Ni and Cr, in this study, represented the dominant contributors to soil pollution. The NI values of Ni (5.69) and Cr (2.61) classified the samples as seriously polluted and moderately polluted, respectively. The INI provided a more refined assessment of soil contamination by incorporating the Igeo. The INI values follow the order Ni > Pb > Zn > Cr > Cu. The results showed that for Ni (1.92), Pb (1.87) and Zn (1.01) the samples are classified as moderately contaminated, while for Cr (0.91) and Cu (0.87) as uncontaminated to moderately contaminated.

### 3.7. Results for Human Health Risk Indices

The risk to residents was calculated for three main sub-populations: men, women and children. As stated in Section 2.7, the relative distributions of key parameters (i.e., body weight, ingestion rate, inhalation rate etc.) were considered. For the non-carcinogenic risk, the HI indices were very low for men and women with no probable exceedance of the value of 1. For children the mean risk value (HI) was also low (0.398); however, there was a possible exceedance of the value of 1 for 8.91% of cases. The risk was mainly driven by Cr and then by Ni (Table 7A). The carcinogenic risk was deemed low and below the threshold value of 10^−4^ for all sub-populations (Table 7B). It is noted that the most conservative RfD value for Cr corresponding to Cr(VI) was used, and, as such, there is a possible overestimation of risk.

### 3.8. Potential Ecological Risk Index Evaluation

The detailed results of Eri and PERI are illustrated in Table S12. Mean, minimum and maximum of Eri and PERI are presented in Table 8. Among the PTEs studied, Ni exhibited the highest Eri values, ranging from 22.97 to 29.79, with a mean value of 27.00. Cr, Cu, Pb and Zn showed lower mean levels, with mean Eri of 4.24, 3.87, 1.95 and 0.86 respectively. According to the detailed results (Table S12) the values of all the PTEs belong to the low-risk category.

The PERI values varied from 29.00 to 42.06, with an average value 37.91, indicating a low ecological risk for the study area. The higher contribution of Ni to the total ecological risk suggests that it is the main factor influencing the ecological risk index, although the overall risk remains low.

### 3.9. Experimental Results

#### 3.9.1. PTEs Soil Content Under the Different Treatments

**Figure 5:**
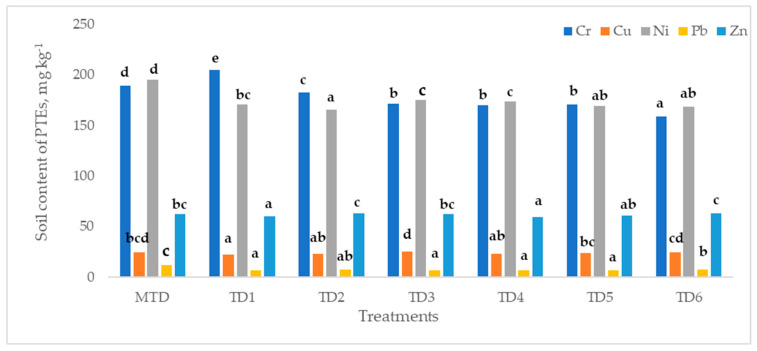
Results of the PTEs content in soil under the different treatments. Different low letters at each column denote statistically significant difference in mean values of the PTEs under the different treatments according to the LSD test with a 95% significance level (*p* < 0.05). Letters are assigned in ascending order of mean values.

The concentration of PTEs in soil under the different treatments after the harvest are presented in Figure 5. Cr and Ni exhibited the highest concentrations among the studied elements, ranging from 159.07 to 205.10 mg kg^−1^ and from 165.50 to 195.46 mg kg^−1^. The highest content of Cr was observed in TD1, followed by a gradual decrease toward TD6. In case of Ni the better decrease was noticed in mixture of 50% manure–50% attapulgite (3% mixture/kg soil) treatment (TD2). Cu concentrations varied from 22.40 (TD1) to 25.36 (TD3) mg kg^−1^. Zn and Pb content ranged between 59.64 (TD4) to 62.95 (TD6) mg kg^−1^ and between 6.74 (TD3) to 11.8 (MTD) mg kg^−1^, respectively.

Compared to the control (PTD), all treatments induced reduction in PTEs concentrations at the end of the experiment. For Cr, the post pronounced decrease was observed under TD6 (159.07 mg kg^−1^), corresponding to a reduction of approximately 16% relative to the control (189.45 mg kg^−1^). In case of Cu, TD1 (22.40 mg kg^−1^) exhibited the highest efficiency, achieving a reduction of about 7.6%. Ni content was most effectively reduced under TD2 (165.50 mg kg^−1^), with a decrease of 15.3%. The greatest reduction was achieved in Pb concentration, with TD3 treatment resulting in a reduction of 42.9% compared to the control treatment. Finally, in case of Zn the highest reduction recorded under TD4, corresponding to a decrease of about 4.5%.

#### 3.9.2. PTEs Plant Tissue Content Under Different Treatments

The contents of PTEs in plant tissue under the different treatments are illustrated in Table 9. For Cr, the lowest concentration was recorded in TD3 (2.12 mg kg^−1^), which differed significantly from other treatments, while the control MTD exhibited the highest value (2.68 mg kg^−1^). In the case of Cu, TD3 (7.40 mg kg^−1^) and TD1 (7.83 mg kg^−1^) presented the lowest content. For Ni, TD6 (1.460 mg kg^−1^) exhibited the lowest concentration and was statistically different from almost all the other treatments. Regarding Pb, TD4 showed the higher reduction value (0.19525 mg kg^−1^). Finally, the best efficiency in reduction of Zn content in plant tissue was noted in TD1 treatment.

The greatest relative reductions compared to the control PTD were observed for Cr and Cu in TD3, with a reduction value of 20.9 and 21.1%, respectively. In the case of Ni the reduction in the TD6 treatment was 9.1%. For Pb the highest % decrease was noted for TD4 with a value of 0.51%. Finally, in the case of Zn, TD1 treatment provoked a reduction of 9.4% in comparison with MTD.

#### 3.9.3. Biological Concentration Factor (BCF) and Coefficient of Contamination Level (CCL)

The BCF and CCL are displayed in Table 10.

Concerning BCF, the results indicated noticeable variation among treatments. For Cr, the lowest values were recorded in TD3 (0.01236) and TD1 (0.1238), suggesting a reduction of 12.7% and 12.5% compared to the control (MTD). In contrast, the TD4 (0.01532) treatment exhibited the highest concentration. In case of Cu, TD3 (0.29180) showed the lowest value, with a % decrease of 24.6 compared to control (MTD). For Ni all treatments increased the BCF values compared to MTD (8.22), with TD2 (10.52) presenting the highest content, whereas TD6 (8.66) remained closer to the control (MTD) levels. Regarding Pb, all treatments exhibited higher concentrations than the MTD (0.0166), with TD2 (0.02908) and TD3 (0.02915) showing the highest values, indicating a consistent increase under treatment conditions. Finally, in case of Zn only the TD1 treatment showed a decrease and in comparison, with control (MTD) that reduce was of a value of 5.59%.

Furthermore, according to the results, Ni exhibited markedly elevated BCF values, ranging from 8.22163 to 10.5152, consistently exceeding the threshold (BCF > 1), indicating a strong accumulation capacity and high transfer from soil to plant.

The CCL results demonstrate diverse effects of manure–attapulgite applications on PTE uptake (Table 11). For Cr, the TD1, TD2 and TD3 treatments showed CCL values lower than 1, indicating reduced uptake compared to control (PTD). In the case of Cu, CCL values ranged from 0.758 to 1.158, with most treatments showing values below 1, suggesting reduced uptake, except for TD2. In contrast, Ni, Pb and Zn exhibited CCL values above 1 across all treatments. Ni ranged from 1.051 to 1.260, while Pb showed the highest values, varied from 1.553 to 1.753, indicating enhanced uptake compared to control (MTD). Finally, the CCL values for Zn ranged from 0.950 (TD2) to 1.260 (TD4). All the treatments demonstrated greater uptake relative to the control (MTD), except the TD2 treatment with values close to the threshold limit.

## 4. Discussion

### 4.1. Discussion on PTEs Concentration of the Studied Soil Samples

The concentration of PTEs in the study area showed a clear dominance of Cr and Ni, with values ranging from 65.6 to 180.4 mg kg^−1^ and 133.2 to 172.8 mg kg^−1^, respectively, exceeding all examined soil quality guidelines. In contrast, Cu, Pb and Zn remained within permissible limits. Cr and Ni are often associated with natural background, and t The main reason for the high values (in relation to GLC contamination categories) found in Agia cultivated soils was attributed to the natural background, as usually noted for Cr and Ni [17]. The elevated concentrations in present study may also reflect the combined influence of anthropogenic activities and lithogenic origin associated with ultrabasic rocks. Regarding anthropogenic input, the emissions from nearby industrial operations, such as the aluminum processing facility, could contribute to the observed enrichment through atmospheric deposition [64,65]. Studies have shown that roadside soils are characterized by elevated Pb and Zn concentrations, often exceeding background levels and decreasing with distance from the road, indicating a strong anthropogenic influence [66,67]. In contrast, the low concentrations of Pb (6.8–11.9 mg kg^−1^), Cu (16.5–34.3 mg kg^−1^) and Zn (31.7–70.6 mg kg^−1^) observed in the present study suggest minimal influence of vehicular activities.

PCA analysis verified that there are possible common source(s) for all the PTEs examined (Zn, Cu, Ni, Pb, Cr) as evidenced by their strong co-loading on Component 1. The very high loadings of Zn, Cu and Ni, together with Pb and Cr, indicate a shared origin. The high enrichment of Ni and of Cr may rather be attributed to dominant lithogenic sources associated with ultrabasic rocks, as suggested in [17], especially since the overall input of Pb, Cu and Zn is low here. Component 2, in contrast, represented here a physicochemical soil gradient rather than a direct pollution source. The positive loadings of pH and CaCO_3_, coupled with the negative loading of organic carbon (OC), reflect variations in soil alkalinity and carbonate content versus organic matter levels. Therefore, this component highlights the controlling influence of soil properties on PTEs distribution, rather than their origin.

### 4.2. Discussion on Contamination Factor (CF), Pollution Load Index (PLI) and Geo-Accumulation Index (Igeo) of the Studied Soil Samples

The CF results of this investigation identified Ni as the dominant pollutant, showing considerable contamination among all samples. Cr exhibited moderate to considerable contamination, while Cu, Pb and Zn remained at low levels, suggesting weaker anthropogenic influence or geogenic control. The PLI further confirms the overall contamination status of the study area, with values indicating moderate pollution in the majority of samples. This suggests that, although individual elements such as Cu, Pb and Zn are present at low levels, their combined impact contributes to a measurable degree of environmental pressure. The predominance of Ni contamination, observed in 100% of samples, underscores its important role in driving the overall pollution load. The Igeo results showed that the soil contamination is mainly attributed to the presence of Ni, with all samples categorized as moderately contaminated. In contrast, Igeo values of Cr, Cu and Pb were classified as uncontaminated. Zn indicated a mixed pattern, with most samples ranging from uncontaminated to moderately contaminated. Overall, the Igeo findings confirm that soil contamination is primarily related tο Ni, with minimal contributions from the other elements.

The results of the present study are consistent with previous research on roadside soil contamination, where moderate pollution levels are commonly reported due to traffic-related activities. The CF results identified Ni and Cr as important contributors, reflecting both anthropogenic inputs and geogenic influences, in agreement with studies by Adimalla et al. [68] and Gao et al. [69]. In contrast, other studies such as Ghosh et al. [70] and Ramdani et al. [71] reported Pb and Zn as dominant pollutants, mainly associated with vehicular emissions.

Furthermore, the results of the present study showed both similarities and differences compared to Negahban and Mokarram [72]. In their study, CF indicated low contamination for Ni, Cu and Zn, while Pb showed high contamination; in contrast, our study identified considerable contamination for Ni. Similarly, their Igeo values classified Ni, Cu and Zn as uncontaminated, whereas our results show Ni as moderately contaminated and the other elements as uncontaminated. Regarding PLI, most sites in their research were uncontaminated, while our results indicated moderate pollution in most samples. Overall, both studies confirm roadside pollution, but they differ in dominant elements and contamination levels. Moreover, the results that emerged by Hosseinzadeh et al. [73] identified Cd as the main element for pollution in the study area while in our study the findings showed Ni as the most important pollutant contributor. Both studies reported moderate levels based on PLI. However, while their Igeo results showed moderate contamination for Cu and Zn, our research indicated Ni as moderately contaminated and the other elements uncontaminated.

### 4.3. Discussion on Nemerow Pollution Index (NI) and Improved (Modified) Nemerow Index (INI) of the Studied Soil Samples

The NI and INI provided a comprehensive assessment of soil contamination by integrating the combined effects of multiple PTEs. According to the results, moderate pollution was indicated across most sampling sites, which indicates that soil quality is influenced by the contribution of several elements rather than a single contaminant. Similar findings have been reported in recent studies where the NI has been widely used to evaluate multi-element contamination and to reflect both average and maximum pollution levels, providing a more holistic assessment of soil quality [16,74]. In the current study, Ni and Cr were identified as the main contributors to soil pollution, which is consistent with previous research revealing that specific elements often dominate pollution indices due to their elevated contents and environmental persistence [75,76].

The INI provided a more accurate assessment of soil contamination by incorporating the Igeo. The ranking (Ni > Pb > Zn > Cr > Cu) indicates that Ni is the main contributor to contamination. The moderate levels of Ni (1.92), Pb (1.87) and Zn (1.01) suggest significant anthropogenic influence.

The results of the current study are in partial agreement with those reported by Ramdani et al. [71], where the NI indicated that most roadside soils ranged from moderate to serious contamination levels. In both studies, most samples were classified as moderately polluted, confirming the effect of multiple PTEs on soil quality. However, a key difference lies in the dominant contaminants; while Ramdani et al. [71] identified Pb and Zn as the main contributors, the present study highlights Ni and, to a lesser extent, Cr as the primary drivers of pollution. Additionally, our results indicate a lower overall contamination level, as reflected by lower NI and INI values.

### 4.4. Discussion on Potential Human Risk Evaluation

It is always important to quantify risk to human health deriving from environmental pollutants such as PTEs, in various industrial and commercial settings [77,78]. This can be effectively achieved through the calculation of indices such as HI and TCR [79], with refinements proposed in subsequent guidelines. Out of these recommendations, the use of distributions of inputs rather than the use of single input values has gained considerable attention in recent risk assessments [80]. In the present case, Monte Carlo simulations with 1000 iterations each were used, to calculate the parameters HQ for each PTE, CR for each PTE, and HI and TCR, for men, women and children. This aided in avoiding potential overestimation or underestimation biases due to fixed values for toxicity response coefficients, background levels or risk exposure parameters [80]. The results showed that there was a small but quantifiable exceedance of the risk threshold for 9% of possible cases for the non-carcinogenic risk to children. This finding is in line with conclusions that the soil near highways is seldom pristine [81,82]. It has to be noted however that the highly conservative, worst-case scenario of Cr(VI) speciation was chosen here [83]. In many natural systems, Cr(VI) often represents only a small fraction of the total Cr. Nevertheless, in industrially impacted areas such as the present one with the aluminum factory, Cr(VI) maybe introduced directly to the environment while chromium speciation may also shift with changing redox or environmental conditions [84,85].

Therefore, there is an apparent discrepancy between the exceedance of international soil quality guidelines for Cr and Ni and the small but non-negligible outcome of the risk assessment. However, soil quality guidelines (e.g., Canadian and Dutch values) are conservative screening thresholds based solely on total concentrations and are designed to flag potential contamination. As such they do not account for site-specific exposure conditions, metal speciation or bioavailability. In contrast, human health risk assessment incorporates exposure pathways (ingestion, inhalation and dermal contact), toxicity reference values and population-specific parameters, providing a more realistic estimate of actual risk [86,87]. Recent studies further support this distinction. For example, a probabilistic risk assessment study showed that although several metals exceeded background or guideline values, the calculated non-carcinogenic risk (HI) remained below acceptable thresholds under realistic exposure conditions [88]. Similarly, a comprehensive assessment of urban soils demonstrated that health risk evaluation depends strongly on exposure pathways and population characteristics rather than concentration level alone [89]. Moreover, recent research highlights that incorporating bioavailability or inaccessibility into risk assessment significantly improves the accuracy of exposure estimation, reinforcing that total concentrations alone are insufficient indicators of health risk [90,91]. Therefore, exceedance of guideline values should be interpreted as an indicator of potential contamination rather than direct health risk [68].

### 4.5. Discussion on Potential Ecological Risk Evaluation

The ecological risk assessment indicated that all PTEs pose a low risk, as reflected by both Er and PERI values. Ni showed the highest contribution to ecological risk, although it remained within the low-risk category, while Cr, Cu, Pb and Zn exhibited minimal impact. PERI values confirm low ecological risk in the study area, which means that the elemental concentrations are limited and do not pose significant environmental concern. The Er values of the current study are in agreement with those reported in the research by [92], where most elements indicated low ecological risk (Er < 40) and PERI values were within the low-risk classification (RERI < 95). The Er and PERI results of our study differ from those of Kholikulov et al. [93]. Their study reported high pollution levels for Er and PERI values and this pollution is attributed to Pb and Zn, while ours showed low risk, with Ni as the main contributor. Moreover, the investigation by Rana et al. [94] reported a different pattern, where Cd posed moderate ecological risk, while the other studied elements showed low risk. Here there was also a discrepancy between some international soil guidelines that were substantially exceeded and the “low-risk” ecological outcome, based on PERI calculations. Again, this discrepancy likely reflects methodological differences between contamination-based ecological indices and precautionary guideline-based protection thresholds.

### 4.6. Discussion on PTEs Soil and Plant Content Under the Different Treatments

The results demonstrated that the application of organic and mineral amendments significantly influenced the concentration and behavior of PTEs in both soil and plant tissues. Among the studied elements, Cr and Ni exhibited the highest content in soil samples, suggesting either a geogenic background or additional anthropogenic inputs. The observed decrease in Cr concentration from TD1 to TD6 indicates that increasing amendment effectiveness enhanced immobilization processes, likely through adsorption onto attapulgite surfaces and complexation with organic matter [95]. Similarly, the most effective reduction of Ni under TD2 suggests that the combined application of manure and attapulgite at specific rations can improve stabilization mechanisms, although Ni remained elevated due to its higher mobility compared to other elements [95,96].

Cu, Zn and Pb showed lower concentrations, indicating limited contamination and stronger retention in the soil matrix. The reduction of Cu and Zn under TD1 and TD4, respectively, suggest that these elements are partially responsive to amendment application, while the decrease in Pb under TD3 highlights the effectiveness of amendments in immobilizing elements with inherently low mobility. The reduction of Pb (up to 42.9%) is consistent with its strong affinity for soil particles and its tendency to form stable complexes and precipitates which limit its availability [97,98].

The analysis of plants content further supports the effectiveness of the applied treatments. Lower concentrations of Cr and Cu in TD3 indicate enhanced immobilization and reduced bioavailability. In contrast, Ni showed a smaller reduction in plant tissue, even under the most effective treatment (TD6), confirming its high mobility and bioavailability in soil–plant systems [99,100]. Pb exhibited minimal accumulation in plant tissues across all elements, despite its presence in soil, reflecting its limited translocation capacity [101]. Zn showed moderate variability, with TD1 demonstrating the highest reduction in plant uptake [100,102].

Overall, the results indicate that the applied treatments were effective in reducing both soil content and plant uptake of PTEs, although their efficiency varied among elements. The greatest reduction was observed for less mobile elements such as Pb and Cr, while more mobile elements such as Ni were less affected. This highlights the importance of element-specific behavior in determining remediation efficiency [24,32].

Furthermore, the combined use of manure and attapulgite appears to enhance immobilization processes through synergetic mechanisms, including adsorption, complexation and precipitation. These findings confirm that soil amendments can play a crucial role in reducing metal bioavailability and improving soil quality, thereby contributing to safer agricultural production systems and mitigating the transfer of PTEs into food chain [24,32].

Moreover, the results of this study are consistent with recent studies demonstrating that soil amendments can significantly reduce toxic elements mobility and bioavailability by transforming them toward less available forms [20,24,32,95]. In particular, attapulgite and organic materials have been shown to decrease bioavailable metal fractions and promote their conversion into stable forms, thereby limiting plant uptake [20,30,35,95].

### 4.7. Discussion on Biological Concentration Factor (BCF) and Coefficient of Contamination Level (CCL)

The BCF results revealed clear differences in the accumulation behavior of the studied elements. Ni showed high BCF values (>1), indicating strong plant uptake and efficient transfer from soil to plant, in agreement with recent studies highlighting its high mobility and bioavailability in agricultural soils [25,103]. When combined with CF results, Ni exhibited both the highest CF values (mean 5.40) and elevated BCF values (>8), highlighting it as the most critical element in terms of environmental and food safety risk. In contrast, Cr and Cu showed low BCF values, reflecting limited bioavailability, despite Cr presenting moderate to considerable CF levels. Cr and Cu presented low BCF values because they are strongly bound to soil constituents and occur in less soluble forms, resulting in limited bioavailability despite elevated soil concentrations [104,105]. Pb exhibited low CF and BCF values, confirming its low mobility and restricted plant uptake. Zn showed low contamination (CF < 1) but moderate BCF behavior, indicating its higher mobility compared to Pb and Cu. This pattern is consistent with previous studies, where limited soil–plant transfer of Pb has been reported due to its strong binding to soil particles. On the other hand, Zn is more bioavailable and readily taken up by plants, even at low soil concentrations [100,106]. The comparison between CF and BCF demonstrates that high soil concentrations do not necessarily result in increased plant uptake, emphasizing the importance of element-specific properties and soil conditions in controlling bioavailability [107,108].

The CCL results indicate that manure–attapulgite application exerts element-specific effects on PTE uptake, reflecting differences in metal mobility and bioavailability. For Cr, the consistently lower CCL values (<1) across TD1, TD2 and TD3 suggest that the applied amendments effectively reduced its bioavailability, likely through immobilization mechanisms such as adsorption onto mineral surfaces or precipitation, which are commonly associated with clay minerals and attapulgite-based amendments [109]. A similar, though less pronounced, trend was observed for Cu, where most treatments resulted in reduced uptake (CCL < 1), except for TD2, indicating that amendment composition rate may influence Cu dynamics and adsorption behavior in soil systems [6].

In contrast, Ni, Pb and Zn exhibited CCL values greater than 1 across most treatments, indicating that their uptake was not suppressed and, in some cases, it was enhanced relative to the control. This outcome can be attributed to the element-specific behavior and mobility of these PTEs in soil–plant systems. In particular, Ni and Zn are known to exhibit relatively higher mobility and bioavailability compared to other elements, which facilitates their uptake by plants even under amended conditions [110,111]. The particularly high CCL values observed for Pb, despite its typically low mobility, suggest that localized changes in soil chemical conditions or rhizosphere processes may have influenced its availability. Additionally, the incorporation of organic amendments may enhance metal solubility through the formation of soluble organo-metal complexes and changes in soil chemical conditions, thereby temporarily increasing the availability of certain elements for plant uptake [6,112]. In the case of Pb, although this element is generally characterized by relatively low mobility in soils, the observed increase in CCL values may be related to rhizosphere-induced mobilization processes and competitive interactions among elements affecting uptake dynamics [6,113,114].

Overall, these findings demonstrate that while manure–attapulgite amendments can reduce the uptake of certain elements, their effectiveness is not uniform across all PTEs. This underscores the importance of considering element-specific behavior when evaluating amendment strategies for contaminated soils.

## 5. Conclusions

This study provides a first-line assessment of PTEs contamination in agricultural soils impacted by anthropogenic and lithogenic enrichment and it evaluates the effectiveness of combined organic-mineral amendments for possible remediation of this pollution. The present results demonstrated that Cr and Ni were the dominant elements in the study area, exceeding international soil quality guidelines, while Cu, Pb and Zn remained within acceptable limits. Pollution indices (CF, PLI, Igeo, NI and INI) consistently identified Ni as the main element to contamination and the overall pollution level classified as moderate. Ecological risk assessment indicated that all elements posed low risk, with PERI values below critical thresholds. This discrepancy reflects methodological differences between contamination-based ecological indices and precautionary guideline-based limits.

The origin of these PTEs is mostly lithogenic for Ni and Cr, with anthropogenic components possibly due to the high velocity traffic and/or the adjacent aluminum-based industry. Statistical analysis showed concomitant fluctuations of PTEs along the 13 samples pointing toward common sources.

The application of manure and attapulgite was effective in reducing PTE content in soil and limiting their uptake by wheat plants. The treatments significantly decreased metal availability, particularly for less mobile elements such as Pb and Cr, while more mobile elements like Ni showed lower reduction efficiency. Plant uptake results and BCF analysis further confirmed that metal accumulation is element-specific, with Ni showing strong transfer from soil to plant, indicating potential food safety concerns.

Overall, the findings highlight that agricultural fields may be significantly burdened with PTEs. The combined use of manure and attapulgite represents a promising, sustainable strategy for mitigating toxic metal mobility and reducing risks to agricultural systems and human health. Future research should be focused on long-term field applications and the optimization of amendment ratios to further enhance remediation efficiency.

## Figures and Tables

**Table 1:** Treatments of the greenhouse experiment.

Code	Treatment
ΜTD (control)	4 kg soil plus *Triticum durum*
TD1	4 kg soil plus mixture of 25% manure–75% attapulgite (3% mixture/kg soil) plus *Triticum durum*
TD2	4 kg soil with plus mixture of 50% manure–50% attapulgite (3% mixture/kg soil) plus *Triticum durum*
TD3	4 kg soil plus mixture of 75% manure–25% attapulgite (3% mixture/kg soil) plus *Triticum durum*
TD4	4 kg contaminated soil with the highest heavy metals concentration plus mixture of 25% manure–75% attapulgite (4% mixture/kg soil) plus *Triticum durum*
TD5	4 kg soil plus mixture of 50% manure–50% attapulgite (4% mixture/kg soil) plus *Triticum durum*
TD6	4 kg soil n plus mixture of 75% manure–25% attapulgite (4% mixture/kg soil) plus *Triticum durum*

**Table 2:** Background values of the examined PTEs.

Cr	Ni	Cu	Zn	Pb
mg kg^−1^				
59.5	29	38.9	70	27

**Table 3:** Values of physicochemical properties of the collected soil samples.

	pH	Electrical Conductivity	CaCO_3_	Organic Matter	Sand	Clay	Silt
	-	(μS/cm)	(%)				
Range	7.1–8.3	277–662	1–5.5	1.2–2.6	17–37	17–53	12–45
Mean	7.8	488.5	2.1	1.7	26.2	42.2	31.6

**Table 4:** Average, minimum and maximum concentration of the measured PTEs.

		Cr	Cu	Ni	Pb	Zn
		mg kg^−1^				
n = 13	Maximum	180.4	34.3	172.8	11.9	70.6
	Minimum	65.6	16.5	133.2	6.8	31.7
	Average	126.1	30.1	156.6	10.5	60.0
	Limits	Cr	Cu	Ni	Pb	Zn
	Canadian SQGs (for agricultural soils) (Canadian Council of Ministers of the Environment 2026) [60]	64	63	45	70	250
	Dutch Target Values [61]	100	36	35	85	140
	Greek limit values (worst-case, pH < 6) [62]	50	40	30	50	100
	Kabata-Pendias [42]	59.5	38.9	29	27	70
	Haynes 2016 [63]	100	60	84	14	70
	Skordas and Kelepertsis [17]	299 (40–25,000)	73 (14–427)	189 (17–1461)	15 (5–60)	87 (39–179)

**Table 5:** Average, minimum and maximum of contamination factor (CF) and Pollution Load Index (PLI) results.

	Contamination Factor (CF)							PLI	
	Unitless								
			Cr	Cu	Ni	Pb	Zn		
n = 13	Maximum		3.03	0.88	5.96	0.44	1.009	1.387	
	Minimum		1.10	0.42	4.59	0.25	0.453	0.755	
	Average		2.119	0.773	5.400	0.390	0.857	1.237	
	CF	Class I <1 Low contamination	Class II 1–3 Moderate contamination		Class III 3–6 Considerable contamination		Class IV >6 Very high contamination		
	PLI	Class I <0.7 unpolluted	Class II 0.7–1 slightly polluted		Class III 1–2 moderately polluted		Class IV 2–3 severely polluted		Class V >3 heavily polluted

**Table 6:** Average, minimum and maximum of Geo-accumulation Index (Igeo)results.

			Igeo_Cr_	Igeo_Cu_	Igeo_Ni_	Igeo_Pb_	Igeo_Zn_
			Unitless				
Maximum			−0.5850	−0.7665	1.9900	−1.7670	1.2363
Minimum			−2.0466	−1.8223	1.6145	−2.5743	0.0215
Average			−1.1473	−0.9763	1.8458	−1.9553	0.7112
Class attributed			0	0	2	0	0
Igeo	Class 0 Igeo ≤ 0 Uncontaminated	Class 1 0 < Igeo ≤ 1 From Uncontaminated to Moderately Contaminated	Class 2 1 < Igeo ≤ 2 Moderately Contaminated	Class 3 2 < Igeo ≤ 3 From Moderately Contaminated to Strongly Contaminated	Class 4 3 < Igeo ≤ 4 Strongly Contaminated	Class 5 4 < Igeo ≤ 5 From strongly Contaminated to Extremely Contaminated	Class 6 5 < Igeo ≤ 10 Extremely Contaminated

**Table 7:** HI (**A**) and TCR (**B**) percentiles for men, women and children.

**(A)**	**Exposed Population**		
**Percentiles**	**Men**	**Women**	**Children**
50%	0.041	0.050	0.269
75%	0.085	0.096	0.572
90%	0.136	0.153	0.925
Mean value	0.058	0.066	0.398
Limit exceedance	-	-	**8.91% > 1**
**(B)**	**Exposed Population**		
**Percentiles**	**Men**	**Women**	**Children**
50%	7.6 × 10^−7^	7.9 × 10^−7^	9.1 × 10^−7^
75%	2.2 × 10^−6^	2.5 × 10^−6^	2.7 × 10^−6^
90%	3.8 × 10^−6^	4.6 × 10^−6^	5.0 × 10^−6^
Mean value	1.2 × 10^−6^	1.4 × 10^−6^	1.5 × 10^−6^
Limit exceedance	-	-	-

Values in bold are above the risk threshold.

**Table 8:** Average, minimum and maximum of Eriand PERI results.

	Eri					PERI
	Unitless					
	Cr	Cu	Ni	Pb	Zn	
Maximum	6.06	4.41	29.79	2.15	1.01	42.06
Minimum	2.20	2.12	22.97	1.26	0.45	29.00
Average	4.24	3.87	27.00	1.95	0.86	37.91

**Table 9:** Results of the PTEs content in plant tissue under the different treatments.

Code	Cr	Cu	Ni	Pb	Zn
	mg kg^−1^				
ΜTD	2.68 e	9.38 d	1.607 b	0.19685 ab	20.23 b
TD1	2.54 de	7.83 ab	1.597 b	0.19700 ab	18.32 a
TD2	2.38 bc	10.38 e	1.740 c	0.20750 b	23.41 cd
TD3	2.12 a	7.40 a	1.614 b	0.19625 ab	24.89 e
TD4	2.60 de	8.88 c	1.580 ab	0.19525 ab	24.25 de
TD5	2.45 cd	8.18 b	1.595 b	0.19675 a	24.42 de
TD6	2.28 b	9.25 cd	1.460 a	0.19700 ab	22.51 c

Different low letters at each column denote statistically significant difference in mean values of the PTEs under the different treatments according to the LSD test with a 95% significance level (*p* < 0.05). Letters are assigned in ascending order of mean values.

**Table 10:** BCF comparison of different treatments.

Code	Cr	Cu	Ni	Pb	Zn
	Unitless				
ΜTD	0.01416 b	0.38712 d	8.22163 a	0.01665 a	0.32454 a
TD1	0.01238 a	0.34969 bc	9.33657 bc	0.02820 bc	0.30639 a
TD2	0.01304 a	0.44623 e	10.5152 d	0.02908 c	0.37219 b
TD3	0.01236 a	0.29180 a	9.21551 bc	0.02915 c	0.39992 c
TD4	0.01532 c	0.38017 d	9.09190 bc	0.02873 c	0.40668 c
TD5	0.01432 b	0.34325 b	9.44461 c	0.02837 bc	0.40072 c
TD6	0.01431 b	0.37495 cd	8.65538 ab	0.02585 b	0.35764 b

Different low letters at each column denote statistically significant difference in mean values of BCF under the different treatments according to the LSD test with a 95% significance level (*p* < 0.05). Letters are assigned in ascending order of mean values.

**Table 11:** CCL comparison of different treatments.

Code	Cr	Cu	Ni	Pb	Zn
	Unitless				
TD1	0.877 a	0.906 b	1.136 ab	1.694 a	0.950 a
TD2	0.918 a	1.190 c	1.301 c	1.690 a	1.138 bc
TD3	0.856 a	0.781 a	1.124 ab	1.721 a	1.221 bc
TD4	1.069 b	0.981 b	1.109 ab	1.689 a	1.225 c
TD5	0.999 b	0.905 b	1.140 b	1.637 a	1.199 bc
TD6	1.016 b	0.990 b	1.022 a	1.557 a	1.070 b

Different low letters at each column denote statistically significant difference in mean values of CCL on different treatments according to the LSD test with a 95% significance level (*p* < 0.05). Letters are assigned in ascending order of mean values.

## Data Availability

The original contributions presented in this study are included in the article/Supplementary Materials. Further inquiries can be directed to the corresponding authors.

## Supplementary Materials

The following supporting information can be downloaded at: https://www.mdpi.com/article/10.3390/toxics14060483/s1, Table S1. A: LOQ/LOD of FAAS for the studied PTEs B: % recovery of the CRM; Table S2. Classification categories of contamination factor; Table S3. Classification categories of Pollution Load Index; Table S4. Classification categories of Geo-accumulation Index; Table S5. Classification categories of Nemerow Pollution Index; Table S6. Classification categories of Improved (modified) Nemerow index; Table S7. Human risk assessment parameters and their distributions; Table S8. RfDs and SFs of the studied PTEs; Table S9. Classification of the single-factor potential ecological risk index and Potential Ecological Risk Index; Table S10. Contamination Factor (CF) values and Pollution Load Index (PLI) for the studied soil samples; Table S11. Igeo index values for the studied soil samples; Table S12. Single-factor potential ecological risk index (Eri) and Potential Ecological Risk Index (PERI). References [115,116,117,118,119,120,121,122,123,124,125,126,127,128,129,130] are cited in the supplementary materials.
