# Supplementary material for: Evaluation of Potentially Toxic Elements in Roadside Agricultural Soils Using Pollution Indices and Remediation Potential of Manure and Attapulgite in Wheat Cultivation
Source: Toxics. 2026 May 31;14(6):483. doi: 10.3390/toxics14060483 (PMC13307833; doi:10.3390/toxics14060483)
Supplement: Supplementary file 1 [file toxics-14-00483-s001.zip › toxics-4291653-supplementary.pdf]

SUPPLEMENTARY MATERIAL

Table S1. A: LOQ/LOD of FAAS for the studied PTEs B: % recovery of the CRM.

	Cr	Cu	Ni	Pb	Zn
LOD (mg/kg soil)	1.10	0.59	0.71	0.60	1.29
LOQ (mg/kg soil)	3.65	1.96	2.37	1.99	4.30

	Cr	Cu	Ni	Pb	Zn
low concentration	98.5	104.8	97.8	92.9	100.4
medium concentration	95.8	91.2	99.6	96.0	91.0
high concentration	94.4	93.3	93.3	93.6	102.9
average	96.2	96.4	96.9	94.2	98.1

Table S2. Classification categories of Contamination Factor (CF)[41] .

Classes	Values	Contamination level
I	<1	Low contamination
II	1 - 3	Moderate contamination
III	3- 6	Considerable contamination
IV	>6	Very high contamination

Table S3. Classification categories of Pollution Load Index (PLI)[115, 116].

Indices	Values	Characterization
I	<0.7	unpolluted
II	0.7 - 1	slightly polluted
III	1 - 2	moderately polluted
IV	2 - 3	severely polluted
V	>3	heavily polluted

Table S4. Classification categories of Geo-accumulation Index (Igeo)[45].

Grades	Igeo Value	Characterization
0	≤ 0	Uncontaminated
1	0<Igeo≤1	Slightly contaminated
2	1<Igeo≤2	Moderately contaminated
3	2<Igeo≤3	From moderately contaminated to strongly contaminated
4	3<Igeo≤4	Strongly contaminated
5	4<Igeo≤5	Seriously contaminated

6	5<Igeo≤10	Extremely seriously contaminated

Table S5. Classification categories of Nemerow Pollution Index (NI) [46] .

Nemerow Pollution Index	Classes
NI < 0.7	safe
0.7 < NI ≤ 1	precaution
1 < NI ≤ 2	slightly polluted
2 < NI ≤ 3	moderately polluted
NI > 3	seriously polluted

Table S6. Classification categories of Improved (modified) Nemerow index (INI)[46,117] .

Improved Nemerow Pollution Index	Classes
INI < 0.5	uncontaminated
0.5 < INI ≤ 1	uncontaminated to moderately contaminated
1 < INI ≤2	moderately contaminated
2 < INI ≤ 3	moderately to heavily contaminated
3 < INI ≤ 4	heavily contaminated
4 < INI ≤ 5	heavily to extremely contaminated
INI > 5	extremely contaminated

Table S7. Human risk assessment parameters and their distributions.

		**Residents**				
parameter	units	children	female	male	Probabilistic distribution	Source
PTE concentration	mg/kg soil	mean measured concentrations± SDEV			Lognormal[a]	Present data
Average body weight (BW)	kg	50th:22<br>95th:28	66.5 ±13<br>(mean ±SD)	79.7 ±13<br>(mean ±SD)	Lognormal[a]	[52-56]
Exposure duration (ED)	year	from 0 to 6	from 0 to 24	from 0 to 24	Uniform[a]	[118]
Exposure frequency (EF)	day/year	350 (180, 365)	350 (180, 365)	350 (180, 365)	Triangular[a]	[118]
Conversion factor (CF)	-	1 x 10^{-6}			Point[a]	[118]
Average time (AT)	day	365 × ED (noncarcinogenic) X24=8760, X6=2190<br>365 × 70 (carcinogenic)=25550			Point[a]	[118]
Skin area exposed to soil (SA)	cm^2	50th: 2373<br>95th: 2822	50th: 6032<br>95th:7312		Normal[a]	[119]

Skin adherence factor (AF)	mg/(cm^{2}d)	0.2 (0, 3.3)	0.07 (0, 0.3)	0.07 (0, 0.3)	Beta-PERT[a]	[118]
Ingestion rate (IngR)	mg/day	50th:60<br>95th:200	50th:30<br>95th:100	50th:30<br>95th:100	Normal[a]	[120]
Inhalation rate (InhR)	m^3/day	50th: 9<br>95th: 13.4	50th:12.6<br>95th: 16	50th: 15.6<br>95th: 19.9	Normal[a]	[121]
Dermal adsorption factor (ABS)		Individual values			Point[a]	[122]
Particle emission factor (PEF)	m^3/kg	1.36×10^{9}			Point[b]	[118]

a [58], b [123]

Table S8. RfDs and SFs of the studied PTEs.

	RfDing	RfDinh	RfDderm	SFing	SFinh	SFderm
Cr(VI)	a) 9×10^{-4}	a) 3×10^{-5}	d) 1.2×10^{-4}	b) 1.6×10^{-1}	c) 51	-
Pb	d) 3×10^{-3}	d) 3.5×10^{-3}	d) 5.25×10^{-4}	d) 8.5×10^{-3}	-	-
Ni	a) 2×10^{-2}	b) 1.4×10^{-5}	b) 5.4×10^{-3}	-	c) 0.9	-
Cu	b) 4×10^{-1}	d) 4.2×10^{-2}	d) 1.2×10^{-2}	-	-	-
Zn	a) 3×10^{-1}	d)3×10^{-1}	e) 3×10^{-1}	-	-	-

a) [124], b)[125], c) [126], d) [127], e) [128].

Table S9. Classification of the single–factor potential ecological risk index (Eri) and Potential Ecological Risk Index (PERI).

**Er**					
Eri < 40<br>Low risk	40 ≤ Eri < 80<br>Moderate risk	80 ≤ Eri < 160<br>Considerable risk	160 ≤ Eri < 320<br>High risk	Eri ≥ 320<br>Very high risk	[129]
PERI					
PERI < 150<br>Low ecological risk	150 ≤ PERI < 300<br>Moderate ecological risk	300 ≤ PERI < 600<br>High ecological risk	PERI ≥ 600<br>Significantly high ecological risk		[130]

Table S10. Contamination Factor (CF) values and Pollution Load Index (PLI) for the studied soil samples.

Sample	Cd	Cr	Cu	Ni	Pb	Zn	PLI
1	ND	3.03	0.84	5.49	0.37	0.890	1.062
2	ND	2.19	0.84	5.51	0.41	1.009	1.060
3	ND	2.38	0.88	5.96	0.43	0.956	1.068
4	ND	2.88	0.82	5.51	0.39	0.857	1.060
5	ND	2.12	0.81	5.51	0.44	0.954	1.057
6	ND	1.81	0.82	5.49	0.42	0.897	1.046
7	ND	1.10	0.42	4.59	0.25	0.453	0.945
8	ND	1.41	0.69	5.21	0.42	0.744	1.019
9	ND	1.87	0.72	5.17	0.39	0.874	1.035
10	ND	2.10	0.75	5.38	0.36	0.854	1.039
11	ND	2.22	0.78	5.45	0.42	0.857	1.050
12	ND	2.44	0.82	5.41	0.41	0.857	1.056
13	ND	2.02	0.85	5.52	0.36	0.933	1.048
Average		**2.119**	**0.773**	**5.400**	**0.390**	**0.857**	**1.030**

Table S11. Igeo index values for the studied soil samples.

Sample	**$Igeo_{Cd}$**	**$Igeo_{Cr}$**	**$Igeo_{Cu}$**	**$Igeo_{Ni}$**	**$Igeo_{Pb}$**	**$Igeo_{Zn}$**
1	ND	-0.5850	-0.8443	1.8708	-2.0324	0.9146
2	ND	-1.0554	-0.8310	1.8763	-1.8544	1.2363
3	ND	-0.9354	-0.7665	1.9900	-1.8038	0.8914
4	ND	-0.6596	-0.8712	1.8772	-1.9613	0.7312
5	ND	-1.1016	-0.8940	1.8781	-1.7670	1.2363
6	ND	-1.3278	-0.8712	1.8718	-1.8289	1.1840
7	ND	-2.0466	-1.8223	1.6145	-2.5743	0.8254
8	ND	-1.6946	-1.1225	1.7964	-1.8289	0.0215
9	ND	-1.2856	-1.0490	1.7859	-1.9475	0.0663
10	ND	-1.1142	-0.9988	1.8425	-2.0768	0.8392
11	ND	-1.0356	-0.9407	1.8608	-1.8416	0.1865
12	ND	-0.9001	-0.8667	1.8517	-1.8544	0.7013
13	ND	-1.1731	-0.8135	1.8790	-2.0471	0.4114
Average		**-1.1473**	**-0.9763**	**1.8458**	**-1.9553**	**0.7112**

Table S12. Single–factor potential ecological risk index (Eri) and Potential Ecological Risk Index (PERI).

	**Eri**						**PERI**
Sample	Cd	Cr	Cu	Ni	Pb	Zn	
1	ND	6.06	4.18	27.43	1.83	0.89	40.40
2	ND	4.38	4.22	27.53	2.07	1.01	39.21
3	ND	4.76	4.41	29.79	2.15	0.96	42.06
4	ND	5.76	4.10	27.55	1.93	0.86	40.19
5	ND	4.24	4.04	27.57	2.20	0.95	39.00
6	ND	3.62	4.10	27.45	2.11	0.90	38.18
7	ND	2.20	2.12	22.97	1.26	0.45	29.00
8	ND	2.81	3.44	26.05	2.11	0.74	35.16
9	ND	3.73	3.62	25.86	1.94	0.87	36.04
10	ND	4.20	3.75	26.90	1.78	0.85	37.48
11	ND	4.44	3.91	27.24	2.09	0.86	38.54
12	ND	4.87	4.11	27.07	2.07	0.86	38.99
13	ND	4.03	4.27	27.59	1.81	0.93	38.63
Average		**4.24**	**3.87**	**27.00**	**1.95**	**0.86**	**37.91**
